# The Role of circHIPK3 in Tumorigenesis and Its Potential as a Biomarker in Lung Cancer

**DOI:** 10.3390/cells13171483

**Published:** 2024-09-04

**Authors:** Eryk Siedlecki, Piotr Remiszewski, Rafał Stec

**Affiliations:** 1 Department of Oncology, Medical University of Warsaw, 02-097 Warsaw, Poland; piotrremiszewski789@gmail.com (P.R.); drrafals@wp.pl (R.S.); 2 Faculty of Medicine, Medical University of Warsaw, 02-091 Warsaw, Poland

**Keywords:** lung cancer, circular RNAs, circHIPK3, tumorigenesis, biomarker, miRNA, progression, expression, metastasis, cell proliferation

## Abstract

Lung cancer treatment and detection can be improved by the identification of new biomarkers. Novel approaches in investigating circular RNAs (circRNAs) as biomarkers have yielded promising results. A circRNA molecule circHIPK3 was found to be widely expressed in non-small-cell lung cancer (NSCLC) cells, where it plays a crucial role in lung cancer tumorigenesis. CircHIPK3 promotes lung cancer progression by sponging oncosuppressive miRNAs such as miR-124, miR-381-3p, miR-149, and miR-107, which results in increased cell proliferation, migration, and resistance to therapies. Inhibiting circHIPK3 has been demonstrated to suppress tumour growth and induce apoptosis, which suggests its potential use in the development of new lung cancer treatment strategies targeting circHIPK3-related pathways. As a biomarker, circHIPK3 shows promise for early detection and monitoring of lung cancer. CircHIPK3 increased expression levels in lung cancer cells, and its potential link to metastasis risk highlights its clinical relevance. Given the promising preliminary findings, more clinical trials are needed to validate circHIPK3 efficacy as a biomarker. Moreover, future research should determine if the mechanisms discovered in NSCLC apply to small cell lung cancer (SCLC) to investigate circHIPK3-targeted therapies for SCLC.

## 1. Introduction

Lung cancer is the most commonly diagnosed cancer worldwide, accounting for 12.4% of the total cases. It is the most frequently diagnosed form of cancer in men and the second most frequently diagnosed in women [1]. Furthermore, it stands as the foremost cause of cancer-related fatalities, as it represents as much as 18.7% of the total cancer deaths [1].

Based on the structure of cancerous cells, lung cancer is divided into two types: small-cell lung cancer (SCLC) and non-small-cell lung cancer (NSCLC) [2]. Non-small cell lung cancer (NSCLC), with adenocarcinoma and squamous cell carcinoma as the most common histological subtypes, is particularly widespread as it constitutes around 80–85% of all lung cancer cases [3]. Adenocarcinomas, whose cells resemble glandular cells and sometimes produce mucin, account for approximately 40% of lung cancers [4]. Squamous-cell carcinomas, representing around 30% of lung cancers, consist of sheets of cells with keratin layers and often have a hollow cavity associated with cell death [3]. They are often found near large airways [3]. Most NSCLC cases are diagnosed at advanced stages (IIIB/C or IV), making treatment more challenging [5]. However, SCLC is thought to be more dangerous as a strong tendency for early metastasis and a poor prognosis characterise it [6]. SCLC tumours are typically located near the centre of the lungs, often in the major airways [7]. The cells are small, with poorly defined borders, minimal cytoplasm, and granular chromatin. They are characterised by their exceptionally high mitotic rates and a tendency to grow in clusters [6].

Recognised environmental factors contributing to lung cancer encompass smoking tobacco, exposure to secondhand smoke, occupational hazards linked to lung carcinogens, radiation exposure, as well as indoor and outdoor air pollution [8]. Cigarette smoking particularly stands out as the primary aetiology of lung cancer and the foremost global contributor to cancer-related deaths [8]. Moreover, the prevalence of smoking has surged in developing countries, initiating lung cancer epidemics within these regions [9]. The link between cigarette smoking and lung cancer is particularly pronounced in the case of SCLC [10].

Given the high morbidity and mortality rates of lung cancer [1], it is necessary to improve the detection and treatment of this disease. It can be achieved by the identification of new biomarkers, which is an acknowledged approach for early disease detection and improving therapies, with the ability to forecast severe side effects and resistance to treatment [11]. Novel approaches in investigating circular RNAs (circRNAs) as biomarkers have shown promising results [12]. Additionally, these molecules have been found to modulate tumour formation and development in malignancies, particularly lung cancer [13].

CircRNA is closely connected to lung cancer; circhipk3 has been associated with processes of tumorigenesis and tumour progression in multiple malignancies, of which its role in these processes in lung cancer stands out in particular [14]. Additionally, some studies indicate its potential role as a promising biomarker in lung cancer diagnostics [15,16].

Therefore, we aim to review the role of circHIPK3 in lung cancer in the process of tumorigenesis and its potential as a biomarker.

## 2. Structure and Biogenesis

CircRNAs are characterised by their lack of 5′ and 3′ terminals, making them more stable with a longer half-life compared to linear RNAs [17]. Certain circRNAs can amass significant concentrations within different cell types, notably in the brain, where they demonstrate high expression levels [18]. Nevertheless, most circRNAs are found to be expressed minimally across various tissues [19]. Despite typically exhibiting lower expression levels compared to linear RNAs, circRNAs emerge as the primary transcript for numerous genes [20].

CircRNAs are a type of endogenous non-coding RNA that is transcribed from linear pre-mRNAs by RNA polymerase II and formed via back splicing, differing from canonical splicing, which forms mRNAs [21]. Depending on their genomic origin, circRNAs can be categorised into four types: exonic circRNAs, circular intronic RNAs, exon–intron circRNAs, and tRNA intronic circRNAs [22,23]. They are conserved, endogenously abundant, and stable in mammalian cells, playing vital regulatory roles in various biological processes and disease occurrences [24,25]. Regarding their functionality, it is believed that circRNA could potentially modulate the targeted regulation of genes that can be regulated by miRNA molecules by serving as miRNA sponges, binding with miRNA, and regulating their function [26]. Moreover, circRNAs can interact with RNA-binding proteins, competing with their parent mRNAs, thereby influencing the translation process and protein function or production [27]. A different way of its operation involves binding, sorting, and sequestering proteins to specific subcellular locations, potentially altering protein–protein interactions [27].

In recent studies, circRNAs have been linked to the process of formation and development of many diseases, including diabetes mellitus [28], inflammatory diseases [29], and cardiovascular diseases [30]. Moreover, they are involved in neuronal injury and neuroinflammation in neurological disorders by regulating miR-124 and miR-638, impacting neuroinflammatory pathways and apoptosis. In ischemic stroke, where the blood supply to part of the brain is interrupted, they alleviate neuronal damage by modulating these miRNAs, reducing infarct size and aiding recovery [31]. Additionally, in neurodegenerative diseases such as Alzheimer’s and Parkinson’s, their modulation of neuroinflammation and apoptosis pathways contributes to neuroprotection and slows disease progression [32]. Furthermore, the role of circRNAs is particularly prominent in various human cancers, especially neoplasms of the lungs, stomach, and gallbladder [19,33,34].

**Figure 1:**
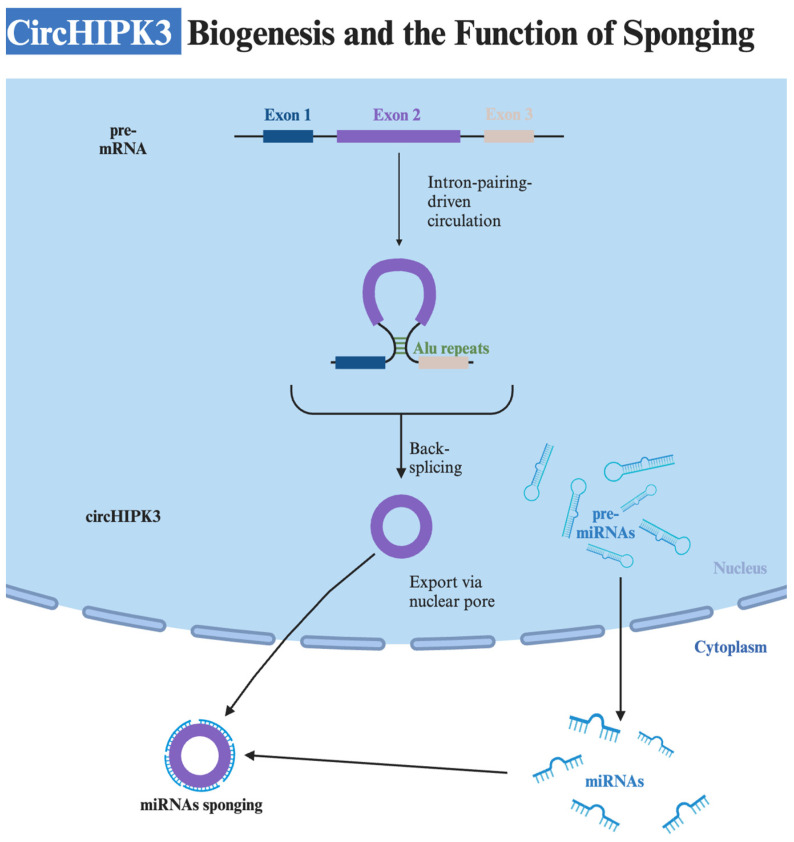
Biogenesis of circHIPK3 involving intron-pairing-driven circularization model and subsequent sponging of miRNAs in the cytoplasm. CircHIPK3 functions as a miRNA sponge, influencing mRNA expression at the post-transcriptional level and thereby promoting or inhibiting progression in various cancers. This circRNA is formed in the nucleus and exported via the nuclear pore to the cytoplasm, where it binds with the miRNAs. The miRNAs are present in the cytoplasm and formed out of pre-miRNAs in the nucleus.

**Figure 2:**
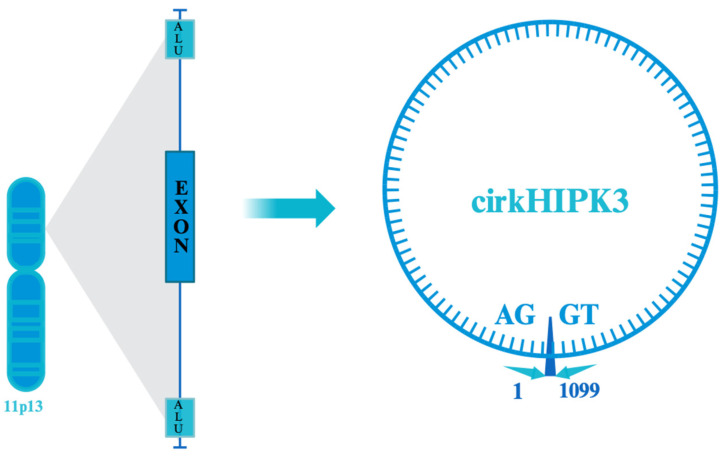
Location on 11p13 chromosome and the formation of circHIPK3 out of exon 2 surrounded by Alu repeats. CircHIPK3 is formed primarily in an intron-pairing-driven circularization model, which is also known as the direct back-splicing mechanism. Reverse complementary sequences flanking introns facilitate the process of back-splicing. These flanking complementary sequences, particularly Alu elements, are essential for exon circularization. Perfectly matched complementary sequences enhance the expression of circRNAs.

The biogenesis of circRNAs occurs through four primary models: lariat-driven circularization, intron-pairing-driven circularization, RNA-binding–protein-binding-driven circularization, and intron-splicing-driven circularization [25,35]. The creation of circHIPK3 primarily involves the intron-pairing-driven circularization model. This model is dependent on ALU elements or flanking inverted repetitive sequences that promote circularization through base-pairing across different introns (Figure 1) [25]. Specifically, the homeodomain-interacting protein kinase 3 gene is located on chromosome 11p13, and its second exon, along with flanking introns with complementary Alu repeats, promotes the formation of circHIPK3 (Figure 2) [18,36,37,38]. Its formation is dependent on canonical spliceosome mechanisms, and the flanking introns mediate the efficiency of circularization [27]. Moreover, cis-acting elements and trans-acting splicing factors like heterogeneous nuclear ribonucleoproteins and SR proteins, as well as dsRNA-specific adenosine deaminase enzymes, play significant roles in its biogenesis [21,39].

## 3. Role of CircHIPK3 in Other Cancers

CircHIPK3 has been discovered to play a crucial role in various non-cancer diseases, and studies have shown the possibility of its clinical application [40]. The main roles of circHIPK3 in non-cancer diseases have been summarised in Table 1.

Moreover, CircHIPK3 has been associated with processes of tumorigenesis and tumour progression in multiple malignancies, and its role in these processes in lung cancer stands out in particular [14]. The general mechanisms of action of circHIPK3 in cancer are summarised in Table 2. CircHIPK3 plays various roles in different cancers by interacting with specific miRNAs and signalling pathways, impacting tumour growth, metastasis, and drug resistance [14]. In in vitro and in vivo experiments, the silencing and overexpression of circHIPK3 had significant effects on the proliferation, migration, and invasion of tumour cells, indicating that expression levels of circHIPK3 are closely related to the biological behaviour of tumour cells [41]. For instance, in colorectal cancer, circHIPK3 sponges miR-7, enhancing focal adhesion kinase and epidermal growth factor receptor expression to promote cell proliferation. In gastric cancer, it targets miR-124 [42], leading to Wnt/β-catenin signalling activation. In breast cancer, circHIPK3 regulates miR-193a, affecting the HMGB1/PI3K/AKT axis [43]. The role and mechanism of circHIPK3 in different cancer types are summarised in Table 3. Currently, due to the technical limitations of available methods, the role of circHIPK3 in cancer and various diseases remains to be further elucidated. MiRNA sponging is one of the primary mechanisms by which circHIPK3 exerts its different functions in various diseases [44].

**Table 1:** Roles of circHIPK3 in non-cancer diseases.

Disease	Role of CircHIPK3	References
Osteoarthritis	Upregulated in osteoarthritis cartilage tissues; silencing promotes chondrocyte apoptosis via miR-124/SOX8.	[40]
Acute Pancreatitis	Promotes pyroptosis via miR-193a-5p/GSDMD axis, aggravating acute pancreatitis; potentially plays a role in disease severity. Potential to be a biomarker for different severe acute pancreatitis stages; important for management of complications like systemic inflammatory response syndrome and multiple organ failure.	[45,46]
Pulmonary Fibrosis	High expression in idiopathic pulmonary fibrosis; induces FMT via miR-338-3p, a potential target for treatment.	[47,48]
Cardiac Fibrosis	Increased expression post-Ang II treatment; associated with cardiac fibrosis; reduced by silencing Ang II.	[49]
Atherosclerosis	Decreased in atherosclerotic mice; suppresses autophagy. Overexpression reverses the inhibition of autophagy by ox-LDL and improves symptoms via the miR-190b/ATG7 pathway.	[50,51,52,53,54,55,56,57]
Asthma	Upregulated in airway smooth muscle cells; promotes proliferation and migration, inhibits apoptosis. Inhibits miR-326, activating STIM1; silences miR-375, upregulating MMP-16.	[58,59]
Age-related Cataract	Downregulated in age-related cataract; inhibits apoptosis and promotes proliferation by targeting miR-221-3p/PI3K/AKT pathways.	[60]
Diabetes Mellitus (DM) and Its Complications	Elevated in Type 2 diabetes mellitus and associated with HbA1c, fasting blood glucose; involved in diabetic nephropathy, cardiomyopathy, neuropathic pain, retinal damage, and aortic endothelial cell proliferation. Sponges various miRNAs (e.g., miR-185, miR-29b-3p, miR-192-5p, miR-124, miR-30a-3p, miR-106a-5p) affecting genes like cyclin D1, PCNA, TGF-β1, Col1a1, Col3a1, FOXO1, VEGF-C, FZD4, WNT2	[61,62,63,64,65]

Abbreviations: AKT, Protein kinase B; Ang II, angiotensin II; ATG7, Autophagy related 7; Col1a1, Collagen, type I, alpha 1; Col3a1, Collagen, type III, alpha 1; CRYAA, Alpha-crystallin A chain; DM, diabetes mellitus; FOXO1, Forkhead box protein O1; FMT, fibroblast-to-myofibroblast transition; FZD4, Frizzled-4; GSDMD, gasdermin D; HbA1c, Haemoglobin A1c; LDL, low density lipoproteins; MMP-16, Matrix metalloproteinase-16; PCNA, Proliferating cell nuclear antigen; PI3K, Phosphoinositide 3-kinase; SOX8, SRY-box transcription factor 8; STIM1, Stromal interaction molecule 1; TGF-β1, Transforming growth factor beta 1; VEGF-C, Vascular endothelial growth factor C; WNT2, Wingless-type MMTV integration site family, member 2.

## 4. The Function of CircHIPK3 in Lung Cancer Tumorigenesis

CircHIPK3 was found to be widely expressed in NSCLC cell lines [86], where it seems to play a crucial role in the lung cancer progression process, as it was determined that forced overexpression of circHIPK3 enhanced the survival and proliferation of lung cancer cells [87]. It was associated with circHIPK3 functioning as a sponge for miR-124 and regulating the expression of miR-124 mRNA targets [87]. MiR-124 levels were found to be decreased in solid cancers, where it acts as a potential tumour suppressor [88]. Mir-124 was found to reduce NSCLC cell proliferation, migration, and metastasis [89,90,91]. Therefore, its sponging by circHIPK3 may have reduced the suppressive character of this molecule. Additionally, overexpression of circHIPK3 significantly increased colony formation in NSCLC cell lines [92]. Colony formation assays measure the ability of cancer cells to grow and form colonies, indicating their potential for sustained growth and resistance to treatment. An increase in colony formation suggests that cancer cells can effectively overcome treatment barriers, facilitating metastasis and recurrence [93].

Moreover, circHIPK3 knockdown was determined to hinder NSCLC cell proliferation, migration, and glycolysis [94]. It was associated with decreased sponging of MiR-381-3p by circHIPK3, leading to the inactivation of the AKT/mTOR signalling pathway [94].

Mir-149 was found to promote NSCLC cells’ autophagy and apoptosis [95]. Through ectopic overexpression of miR-149 using miR-149 mimics, significant inhibition of growth, migration, and invasion of NSCLC cells was observed, mediated through FOXM1. The importance of this process is highlighted by the fact that circHIPK3 is found to regulate miR-149 by sponging [96]. Additionally, it was determined that circHIPK3 acted as a sponge for miR-107, thereby promoting BDNF-mediated cell proliferation and migration. Overexpression of miR-107 inhibited BDNF-mediated NSCLC cell proliferation [86]. These results suggest that circHIPK3 promotes tumour progression in NSCLC by many different pathways, which may provide potential therapeutic targets. 

Induced deficiency of circHIPK3 in NSCLC cells was also observed to result in a notable increase in apoptosis of these cells [87,96], indicating the potential for the possible use of treatments targeting circHIPK3-related pathways to induce apoptosis activation. Therefore, by targeting circHIPK3-related pathways, therapies can specifically induce death in cancer cells, minimising damage to healthy cells as circHIPK3 is particularly prominent in cancer cells, promoting tumour development [86]. This approach can overcome resistance to traditional treatments like chemotherapy and radiation, which cancer cells often develop. Furthermore, therapies that enhance apoptosis can be combined with existing treatments [97].

In a study on C57BL/6 mice, upregulation of exosomal circHIPK3 expression promotes Kras-driven intratumoral heterogeneity, which is necessary for tumour cells to bypass immune surveillance and induce immunosuppression by initiating the infiltration of myeloid-associated tumour macrophages into the lung tumour microenvironment using Kras-driven circRNA signalling. This mechanism leads to immune deregulation and immunosuppression [98]. Moreover, guided lymph node metastasis was observed as a result of this amplified expression of circhHIPK3 [98].

Additionally, circHIPK3 expression may play a role in lung cancer drug resistance. In NSCLC, circHIPK3 was found to sponge miR-124 by sponging, which activity is associated with the process of reversing gefitinib resistance in NSCLC [99]. Moreover, miR-381-3p, another target of circHIPK3, was associated with reducing resistance to anti-programmed cell death 1-based therapy in NSCLC [100]. These findings are highlighted by the previously established role of circHIPK3 promoting drug resistance in gemcitabine treatment of pancreatic cancer [69]. Therefore, circHIPK3 seems to undermine the onco-suppressive activity of certain NSCLC-targeted therapy drugs. 

CircHIPK3 appears to affect multiple miRNAs, which has the effect of exacerbating the progression of NSCLC. These miRNAs and their related circHIPK3 role in NSCLC pathogenesis are listed in Table 4.

These findings indicate that circHIPk3 plays a vital role in tumorigenesis, mainly progression. Consequently, its use as a potential therapeutic target in the treatment of lung cancer should be further investigated, especially in clinical studies. Currently, we were not able to find any ongoing clinical trials that focus on targeting the circHIPK3/miRNA pathway in the treatment of lung cancer. Clinical studies are vital as they directly assess the safety and efficacy of treatments in humans, providing definitive evidence for medical practice. Unlike preclinical studies, clinical trials yield data on how interventions perform in real-world scenarios. This human-centric approach ensures that therapies are not only biologically plausible but also practically effective and safe for patients [101].

There is a noticeable lack of data on the function of circHIPK3 concerning SCLC. This is particularly concerning as SCLC remains a major threat due to its exceptionally high mortality rate [102]. It is possible that circHIPK3 may play a similar role in SCLC, influencing tumorigenesis and resistance to therapies. When it comes to other circRNAs, circVAPA was identified to stimulate SCLC progression [103]. However, circSH3PXD2A was found to inhibit the chemoresistance of SCLC [104]. Nonetheless, it seems likely that the role of circHIPK3 in SCLC would resemble that in NSCLC. Therefore, an important aim of research in the future is to verify to what extent findings from studies on function in NSCLC translate to SCLC.

**Table 4:** MiRNA vital in the NSCLC pathogenesis associated with circHIPK3.

miRNA	Onco-Suppressive Role	Effect of Sponging by CircHIPK3	References
miR-124	Significant suppression of metastasis, active inhibition of NSCLC cell invasion and progression, reduction in cell proliferation, regulation of reversing resistance to gefitinib treatment	Enhancing the survival and proliferation of cancer cells	[87,89,90,91,99]
miR-381-3p	Inhibition of NSCLC cell proliferation, migration, glycolysis, and promotion of apoptosis of lung cancer cells, reduction in resistance to anti-programmed cell death 1-based therapy	Promotion of NSCLC cell proliferation, migration, glycolysis, and reduction in lung cancer cell apoptosis	[94,105]
miR-149	Inhibition of NSCLC cell proliferation and metastasis, promotion of cell autophagy and apoptosis	Reduction in NSCLC cell proliferation, migration and invasion. Reduction in apoptosis	[95,96,106]
miR-107	Inhibition of NSCLC cell proliferation and migration	Promotion of NSCLC cell proliferation and migration	[86]

## 5. Biomarker

Early cancer detection can substantially lower cancer mortality rates and save lives. It is especially important in case of rapidly progressing malignancies such as lung cancer. Consequently, significant efforts have been dedicated to developing new technologies for detecting early indicators of the disease [11]. Biomarkers are cellular, biochemical, or molecular changes in cells, tissues, or fluids that can be measured to detect a disease, indicate its severity, or enable the assessment of pharmacological responses to therapeutic interventions [107]. Identifying new biomarkers is an established approach for early disease detection and improving treatment efficacy. This strategy can also predict severe adverse effects and treatment resistance [108].

As the circHIPK3 was found to be widely expressed in lung cancer cell lines where its overexpression seems to promote lung cancer pathogenesis [86], it may be used as a biomarker. It was already established as a potential biomarker in other various cancers, including gallbladder cancer, cholangiocarcinoma, and osteosarcoma [109]. CircRNAs can be identified in various biological samples, including tissue, extracellular vesicles, and plasma [110]. This makes it easier to measure their levels and potentially use them as a biomarker.

Due to the circHIPK3 expression being significantly upregulated in lung cancer, it was possible to establish receiver operating characteristic curve analysis that enabled discrimination between lung cancer patients and healthy controls, which means that circHIPK3 holds promise in the early detection of the disease. No significant difference was found in circHIPK3 level between genders. Moreover, age did not show a linear correlation with the levels of circHIPK3 [15].

Additionally, it is possible that assessing circHIPK3 levels may be useful in assessing cancer progression. Levels of circHIPK3 are strongly positively correlated with the TNM phase [15]. Assessing the TNM phase in lung cancer is crucial as it helps clinicians choose the most appropriate treatment options and allows the monitoring of treatment progress [111].

Moreover, expression of certain circRNAs was found to be significantly upregulated in lung cancer patients with lymph node metastasis compared to those without [112]. As the guided lymph node metastasis was observed as a result of amplified expression of circhHIPK3 [98], its levels may serve as an indicator of metastasis risk. Assessing the likelihood of cancer spreading to other parts of the body allows for timely interventions, significantly impacting prognosis and treatment strategies. It guides clinicians in tailoring treatments, such as systemic therapies, to target potential metastatic sites. Early detection of metastasis can help in managing symptoms and potentially improve survival rates [113].

Suppressing circHIPK3 has been found to promote autophagy in STK11 mutant lung cancer cell lines (A549 and H838). Furthermore, circHIPK3 and linear HIPK3 (linHIPK3) exert antagonistic effects on autophagy regulation. Thus, researchers suggest that the circHIPK3-to-linHIPK3 ratio (C:L ratio) could serve as a reflection of autophagy levels in cancer cells. Notably, a high C:L ratio (> 0.49) correlates with poor survival, particularly in advanced-stage NSCLC patients. This may be connected with the effect on autophagy regulation [16]. Autophagy is a crucial regulatory factor in cancer development, affecting proliferation, drug resistance, invasion, and metastasis in lung cancer and other tumours [114]. This underscores circHIPK3’s potential clinical utility as a lung cancer diagnostic biomarker when assessing the severity of pathological processes induced by malignancy.

Currently, there are ongoing clinical studies examining circRNA potential as a biomarker in early diagnosis of hepatic cell carcinoma (ID NCT06042842), breast cancer (ID NCT05771337), pancreaticobiliary cancers (ID NCT04584996), and anaplastic large cell lymphoma (ID NCT05934045). However, there are no ongoing clinical studies regarding lung cancer. There is a need to explore this subject in more detail, especially in the clinical setting, as the previous studies on circHIPK3 indicate its potential application as a biomarker in lung cancer.

## 6. Conclusions

Lung cancer, the most diagnosed cancer globally, accounts for the most cancer-related deaths [1]. In order to improve lung cancer detection and treatment, new biomarkers are required, with circRNAs showing promise [12].

CircRNAs regulate gene expression by acting as miRNA sponges and interacting with RNA-binding proteins [26]. They are linked to various diseases, including human cancers, affecting tumour proliferation and migration [33]. Specifically, circHIPK3, from the HIPK3 gene, has been closely associated with lung cancer pathogenesis, highlighting its potential as a diagnostic biomarker [34].

CircHIPK3 plays a crucial role in lung cancer tumorigenesis. In NSCLC, circHIPK3 promotes tumour progression by sponging various oncosuppressive miRNAs such as miR-124, miR-381-3p, miR-149, and miR-107, which play critical roles in cell proliferation, migration, and resistance to therapies [86,87,96,100]. The inhibition of circHIPK3 has been shown to suppress tumour growth and induce apoptosis [94]. This indicates the possibility of developing new treatment strategies using circHIPK3-related pathways as a therapeutic target. These potentially targeted therapeutic methods may allow us to focus more efficiently on cancer cells, limiting the harmful effects of the therapy on healthy cells and overcoming resistance to traditional treatments like chemotherapy and radiation. Additionally, circHIPK3 connection to resistance to established cancer therapies requires further study, as it may provide more effective and precise treatment options.

Despite extensive preclinical research on circHIPK3 in NSCLC, there is a notable gap in our understanding of its role in SCLC, a particularly aggressive form of lung cancer. It is possible that circHIPK3 may play a similar role in SCLC, influencing tumorigenesis and resistance to therapies. Future research should aim to elucidate whether the mechanisms identified in NSCLC apply to SCLC and explore the potential of circHIPK3-targeted therapies in this context.

As a biomarker, circHIPK3 shows promise for early detection and monitoring of lung cancer [16]. Early cancer detection is crucial, particularly for fast-progressing malignancies like lung cancer, as it can significantly reduce mortality rates [1]. Its elevated expression levels in lung cancer cells underscore its potential clinical utility [86]. Moreover, the use of circHIPK3 as a biomarker could be valuable in monitoring lung cancer tumour development, as circHIPK3 levels are strongly correlated with the TNM stage, a key indicator of cancer progression [15]. By tracking circHIPK3 levels, clinicians could potentially more accurately assess tumour advancement, aiding in selecting the most appropriate treatment and evaluating its effectiveness.

Current clinical studies are exploring the potential of circRNAs as biomarkers in other cancers, but similar research is lacking for lung cancer. Given the promising preliminary findings, clinical trials focused on circHIPK3 in lung cancer are urgently needed. Such studies could confirm its efficacy as a biomarker and its role in guiding treatment strategies, ultimately improving patient outcomes.

## 7. Methods

A topic-by-topic literature search was conducted to identify reviews and preclinical and clinical studies concerning the function of circHIPK3 in lung cancer and its potential role as a biomarker in diagnostics. A search for relevant studies was conducted using PubMed, Web of Science, and Scopus for articles published before 1 May 2024. The keywords used for the search regarding lung cancer involved: “circHIPK3” and “lung cancer”. Selection of articles was performed by consensus among all authors. The quality of data in the included studies was assessed using the National Institute of Health (NIH) quality assessment tools. Additionally, we supplemented the review with a description of the circHIPK3 and its role in other cancers based on the most prominent and recent findings in the area.

## Figures and Tables

**Table 2:** General mechanisms of action of circHIPK3 in cancer.

Mechanism	Description	miRNAs Involved	References
Regulation of cell growth	It influences cell cycle regulators and enhances cell proliferation and growth.	miR-124, miR-193a, miR-29b,	[66,67]
Modulation of apoptosis	Controls apoptosis-related proteins, impacting cell survival.	miR-193a	[68]
Influence on metastasis	Affects genes involved in cell migration and invasion, modulating metastatic behaviour.	miR-193a	[43]
Drug resistance	Modulates autophagy (negatively in bladder cancer) and survival pathways, contributing to chemoresistance.	miR-330-5p	[69]

**Table 3:** Role and mechanism of circHIPK3 in different cancer types.

Cancer Type	Role of CircHIPK3	Mechanism of Action	References
Gastric Cancer	Promotes gastric cancer progression; associated with poor prognosis	Sponges miR-124 and miR-29b to regulate COL1A1, COL4A1, and CDK6	[42,67,70]
Colorectal Cancer	Functions as an oncogene; promotes proliferation, migration, and invasion; decreases apoptosis	Sponges miR-7 and miR-1207-5p to regulate FMNL2 expression	[71]
Prostate Cancer	Upregulated in prostate cancer tissues and cells; promotes proliferation and invasiveness	Sponges miR-338-3p to regulate ADAM17 expression	[72,73]
Hepatocellular Carcinoma	Significantly upregulated; promotes proliferation and migration	Sponges miR-124 to regulate AQP3 expression	[74]
Chronic Myeloid Leukaemia	Upregulated in peripheral blood mononuclear cells and serum; promotes progression.	Not specified	[75]
Gallbladder Cancer	Higher expression in cancer cells; inhibits survival and proliferation	Sponges miR-124 to regulate ROCK1 and CDK6	[76]
Osteosarcoma	Downregulated; associated with poor prognosis; suppresses proliferation, migration, and invasion	Not specified	[77]
Glioma	Functions as an oncogene; promotes tumour growth	Sponges miR-124-3p and miR-654, regulating STAT3 and IGF2BP3 expression	[78,79]
Oral Squamous Cell Carcinoma	Upregulated; promotes proliferation	Regulates miR-124	[80,81]
Epithelial Ovarian Cancer	Downregulated; promotes proliferation, migration, and invasion	Not specified	[29]
Nasopharyngeal Carcinoma	Upregulated; promotes proliferation, migration, and invasion	Sponges miR-4288 to regulate ELF3 expression	[82]
Cervical Cancer	Upregulated; promotes cell proliferation and EMT, resulting in tumorigenesis	Sponges miR-338-3p, resulting in upregulation of HIF-1α expression	[83]
Melanoma	Overexpressed in melanoma cells; promotes cell growth and mitigates cell death	Sponges miR-215-5p, upregulating YY1 expression	[36]
Pancreatic Cancer	Upregulated in cancer cells; associated with gemcitabine resistance	Sponges miR-330-5p, upregulating RASSF1, regulating proliferation, invasion, migration, EMT, and apoptosis	[69,84,85]

Abbreviations: ADAM17, A disintegrin and metalloprotease 17; AQP3, Aquaporin 3; CDK6, Cell division protein kinase 6; COL1A1, Collagen, type I, alpha 1; Col4a1, Collagen, type IV, alpha 1; ELF3, E74 like ETS transcription factor 3; EMT, epithelial–mesenchymal transition; FMNL2, Formin-like protein 2; HIF1α, Hypoxia-inducible factor 1-alpha; IGF2BP3, Insulin-Like Growth Factor 2 MRNA Binding Protein 3; RASSF1, Ras association domain-containing protein 1; ROCK1, Rho associated coiled-coil containing protein kinase 1; STAT3, Signal transducer and activator of transcription 3; YY1, Yin Yang 1.

## Abbreviations

ADAM17, A disintegrin and metalloprotease 17; AKT, Protein kinase B; Ang II, angiotensin II; AQP3, Aquaporin 3; ATG7, Autophagy related 7; CDK6, Cell division protein kinase 6; circRNAs, circular RNAs; COL1A1, Collagen, type I, alpha 1; Col1a1, Collagen, type I, alpha 1; Col3a1, Collagen, type III, alpha 1; Col4a1, Collagen, type IV, alpha 1; CRYAA, Alpha-crystallin A chain; DM, diabetes mellitus; ELF3, E74 like ETS transcription factor 3; EMT, epithelial–mesenchymal transition; FMNL2, Formin-like protein 2; FMT, fibroblast-to-myofibroblast transition; FOXO1, Forkhead box protein O1; FZD4, Frizzled-4; GSDMD, gasdermin D; HbA1c, Haemoglobin A1c; HIF1α, Hypoxia-inducible factor 1-alpha; IGF2BP3, Insulin-Like Growth Factor 2 MRNA Binding Protein 3; LDL, low density lipoproteins; linHIPK3, linear HIPK3; MMP-16, Matrix metalloproteinase-16; NSCLC, non-small-cell lung cancer; PCNA, Proliferating cell nuclear antigen; PI3K, Phosphoinositide 3-kinase; RASSF1, Ras association domain-containing protein 1; ROCK1, Rho associated coiled-coil containing protein kinase 1; SCLC, small-cell lung cancer; SOX8, SRY-box transcription factor 8; STAT3, Signal transducer and activator of transcription 3; STIM1, Stromal interaction molecule 1; TGF-β1, Transforming growth factor beta 1; VEGF-C, Vascular endothelial growth factor C; WNT2, Wingless-type MMTV integration site family, member 2; YY1, Yin Yang 1.
